# Role of Health-Consciousness on Purchase Intention of Health and Wellness Food: The Serial Mediating Effect of Attitude and Desire

**DOI:** 10.3390/nu17050746

**Published:** 2025-02-20

**Authors:** Jialiang Pan, Kun-Shan Wu, Di-Man Huang, Shu-Wen Sun

**Affiliations:** 1 School of Business and Management, Jiaxing Nanhu University, Jiaxing 314001, China; 220055@jxnhu.edu.cn; 2 Department of Business Administration, Tamkang University, New Taipei City 251301, Taiwan; eva0225dodo@gmail.com; 3 Department of Business Administration, HungKuo Delin University of Technology, New Taipei City 236302, Taiwan

**Keywords:** health and wellness food, purchase intention, PLS–SEM, Necessary condition analysis, cIPMA

## Abstract

**Background/Objectives**: This study investigates consumers’ purchase intention (PI) toward health and wellness foods (HWF) in China by examining key factors, such as health-consciousness (HC), desire, and attitude toward organic food. **Methods**: Data were collected via online surveys completed by Chinese respondents aged 50–65 years. Of the 270 distributed surveys, 230 valid responses (85.2% effectiveness) were analyzed. A multi-analytic approach was employed, integrating Partial Least Squares–Structural Equation Modeling (PLS–SEM), Necessary Condition Analysis (NCA), and Combined Importance-Performance Map Analysis (cIPMA), to explore the hypothesized relationships. **Results**: The results reveal consumers’ HC has a strong influence on their PI toward HWF, with attitude and desire sequentially mediating this relationship. Furthermore, the results of NCA confirm that HC and desire are necessary conditions for purchasing HWF, whereas attitude is not. **Conclusions**: Based on these findings, the study provides suggestions for future research and practical recommendations for HWF businesses.

## 1. Introduction

In contemporary consumer contexts, the growing emphasis on healthy lifestyles has substantially increased the demand for health and wellness foods (HWF) [1,2,3,4,5,6,7,8,9]. These foods, distinguished by their nutritional value and health benefits, are instrumental in disease prevention and the promotion of healthier living [10,11,12].

The global HWF market was valued at USD 1122.65 billion in 2023 and is projected to reach USD 2104.29 billion by 2032, growing at a compound annual growth rate (CAGR) of 7.23% during the forecast period. The HWF market in China was valued at approximately RMB 328.2 billion (USD 45.5 billion) in 2023 and is expected to expand to RMB 423.7 billion (USD 58.2 billion) by 2032 [13].

HWF, such as dietary supplements, have become particularly popular among older adults [14,15]. In China, 70% of individuals aged between 50 and 65 show interest in dietary supplements tailored toward senior citizens [16]. These trends highlight older consumers (aged 50–65 years) as critical contributors to China’s dietary supplement market. Consequently, it is essential to examine the health-related behaviors of older consumers in China.

The Theory of Planned Behavior (TPB) is among the most extensively applied social psychological models for predicting human behavior and intentions across various contexts, particularly in consumer behavior studies related to HWF [2,4,5]. TPB posits behavior is primarily driven by an individual’s intention to perform the behavior. Intention, which encompasses motivational elements and cognitive planning, is the immediate precursor to the behavior itself. This intention is influenced by three interconnected constructs: attitude, subjective norms, and perceived behavioral control. However, in the context of HWF, purchase intention is often minimally affected by perceived behavioral control [2,17,18]. This may be because the act of purchasing such products generally does not entail significant challenges or complexities. Moreover, subjective norms are criticized for their inconsistent effects on purchase intention, as evidenced in previous studies [2,18,19]. In contrast, attitudes toward HWF are widely regarded as the primary determinant of purchase intention, given that the consumption of these foods tends to be a highly individualistic behavior [2,18]. Based on these considerations, this study excludes perceived behavioral control and subjective norms from the analysis.

The TPB model fails to adequately explain the underlying motivation that drives behavioral intentions. To address this limitation, the Model of Goal-Directed Behavior (MGB) extends the TPB by introducing desire as a key factor providing the motivational force prompting individuals to act [20]. Desire is defined as a strong motivational state characterized by an aspiration or longing for a specific outcome to materialize [21]. It plays a pivotal role in consumer behavior by fostering the motivation necessary to actively pursue preferred outcomes, thereby enhancing the probability of goal attainment [22]. For instance, if an individual aspires to achieve a goal, such as maintaining optimal health, and perceives the purchase of HWF as a means to support this objective, their desire for better health will significantly influence their purchase intention toward HWF. Thus, MGB highlights desire as both a consequence of attitude and a significant predictor of purchase intention toward HWF.

The motivations for consuming HWF are extensively explored in prior research. Existing studies identify various factors influencing consumers’ purchasing decisions, including consumers’ attitude [5,18,23], brand-related attributes (e.g., brand image, quality, satisfaction, and loyalty [7]), desire [24,25], environmental concerns [5], nutritional literacy [6], perceived value [3,5], sociopsychological factors [1,17], trust [8,9], and health-consciousness [3,5,8,18,23]. Among these studies, the key drivers for purchasing HWF are identified as attitude, desire, and health-consciousness [3,5,8,18,23,24,25]. A study guided by the MGB framework, comprising attitude, desire, and intention, examines organic food consumers in Mexico [24]. The study explores the influence of attitude on both desire and purchase intention, with desire mediating the nexus between attitude and purchase intention. It is possible there is a relationship between health-consciousness, attitude, desire, and purchase intention. However, this was not investigated in the aforementioned model.

Collectively, the research gaps identified in the extant literature can be summarized as follows. First, there is limited understanding of mature consumers’ purchase intention toward HWF from the perspective of the simplified MGB, specifically concerning health-consciousness, which is a critical dimension of HWF. Conducting a comprehensive and simultaneous examination of the effects of health-consciousness on attitude, desire, and purchase intention would provide HWF business owners and managers with actionable insights to develop targeted strategies for enhancing consumers’ purchase intention. Second, within the simplified MGB framework, this study investigates the factors that influence desire and also examines their subsequent impact on consumers’ purchase intention toward HWF. This approach advances the current understanding of the antecedents of purchase intention for HWF and elicits the mechanisms driving consumer behavior in this context. Third, existing studies predominantly focus on the additive sufficiency of influencing factors for achieving positive outcomes, often overlooking their necessity. However, a necessary cause must be present or reach a specific threshold for an outcome to occur [26,27]. To address these literature gaps, this study seeks to answer the following research questions:

(1) How does health-consciousness directly influence consumers’ purchase intention and indirectly influence it through the sequential mediating effects of attitude and desire?
(2) How does health-consciousness influence desire via attitude as a mediator?
(3) What factors constitute sufficient and necessary conditions for enhancing consumers’ purchase intention toward HWF?

This study aims to examine the impact of health-consciousness on purchase intention toward health and wellness food (HWF) in China, with a particular focus on the serial mediating roles of attitude and desire. A multi-analytic approach is employed, incorporating Partial Least Squares–Structural Equation Modeling (PLS–SEM), Necessary Condition Analysis (NCA), and Combined Importance-Performance Map Analysis (cIPMA). This research contributes to extant literature and theories and offers several significant insights for enterprise managers. First, this research enriches the existing literature on consumer behavior of the elderly population in China toward HWF by examining the direct impact of health-consciousness on purchase intention and its indirect effects through the sequential mediating roles of attitude and desire. Furthermore, the study explores the mediating effect of attitude on the nexus between health-consciousness and desire. These findings provide actionable insights for HWF companies in China to develop effective strategies for expanding consumer bases by focusing on the interplay between health-consciousness, attitude, desire, and purchase intention. Second, by combining PLS–SEM and NCA, this study adopts a robust methodological framework to analyze both causal pathways and the necessary antecedents of purchase intention. Beyond validating the hypothesized model, this dual-method approach identifies whether health-consciousness, attitude, and desire are necessary conditions for purchase intention. Determining if these factors must meet specific thresholds to influence purchase intention offers valuable strategic insights for HWF managers. This evidence enables the identification of critical bottlenecks in marketing strategies, ultimately driving higher levels of purchase intention.

## 2. Materials and Methods

### 2.1. Research Framework

**Figure 1:**
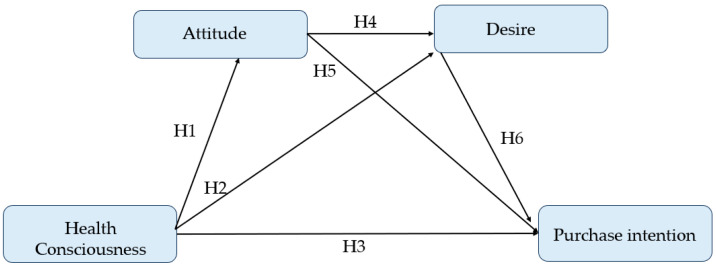
Conceptual framework and study hypotheses.

As discussed in the previous section, this study adopts a simplified perspective of the MGB model [24] as its fundamental theoretical framework, integrating health-consciousness as an additional variable into the research model. This enhancement aims to better elucidate individuals’ behavioral decisions regarding the purchase of health and wellness food. The proposed framework, illustrated in Figure 1, posits that health-consciousness, attitude, and desire collectively influence consumers’ purchase intentions toward health and wellness food (HWF). This integrated approach is innovative in its application and is expected to provide a more nuanced understanding of consumer behavior within this context.

### 2.2. Relationships Between Health-Consciousness, Attitude, Desire, and Purchase Intention

Consumers who incorporate health considerations into their daily routines are described as “health-conscious” [28,29,30]. Health-conscious individuals tend to value not only the health benefits and disease prevention associated with healthy food, but also the contributions to reducing stress, enhancing comfort, and improving life satisfaction [31,32]. As a result, these individuals are more inclined to adopt healthy diets, often selecting food products based primarily on their health benefits [33].

Extensive research demonstrates a strong positive relationship between health-consciousness and consumers’ attitude toward organic food across different cultural contexts [34,35,36]. For example, research [37] emphasizes the critical link between consumers’ health-consciousness and attitude toward their purchase intention for organic food. Establishing consumers’ positive perception is essential, as it has a significant impact on their purchasing and consumption behaviors [38]. Similarly, researchers [39] found health-consciousness significantly influences attitude toward healthy food. This evidence emphasizes the nexus between consumer attitude and their purchase intention toward HWF.

The concept of health-consciousness encompasses more than a mere desire for food; it signifies a comprehensive approach to nutrition, emphasizing the nourishing and healing potential of dietary choices. The inclination toward consuming HWF is driven by an individual’s concern for their personal health, stemming from the perception of HWF as a superior, more nutritionally beneficial option that offers protection against potential health risks. This coincides with the desire for a safer, healthier, and environmentally responsible lifestyle. Studies evidence health-consciousness strongly predicts the desire to travel to resorts [40] and the desire to use wearable health devices [41].

Furthermore, health-consciousness is identified as a significant motivator for consumers to purchase healthy food and supplements [42]. Scholars [5] investigate the factors impacting consumer intention to adopt sustainable healthy dietary patterns in China and confirm consumers’ health-consciousness positively impacts purchase intention toward HWF. Researchers [39] investigating Generation Z’s consumption behavior toward organic food in China suggest that health-consciousness has a positive impact on purchase intention. Research in India [30] also confirms that health-consciousness significantly affects purchase intention, while in Brazil [8] an investigation on consumer purchase intention toward healthy food elicits that health-consciousness significantly and positively influences purchase intention. Considering the aforementioned discussion, the following hypotheses are proposed:

**H1.**  *Consumers’ health-consciousness positively affects attitude toward HWF.*

**H2.**  *Consumers’ health-consciousness positively affects desire toward HWF.*

**H3.**  *Consumers’ health-consciousness positively affects purchase intention toward HWF.*

### 2.3. Relationships Between Attitude, Desire, and Purchase Intention

The Model of Goal-Directed Behavior (MGB) [20] posits that attitude toward a specific object cultivates desire and, in turn, determines whether individuals will form the intention to engage in the corresponding required behavior. In the context of organic food consumption, consumers’ positive attitude toward organic food cultivates a strong desire to purchase it, which subsequently enhances their intention to do so. Furthermore, a direct relationship between attitude and behavioral intention can also be posited. For example, research [24] demonstrates that attitude positively influences both desire and purchase intention toward organic food in Mexico, with desire mediating the relationship between attitude and purchase intention. Other research [43] elicits that consumers’ positive attitudes toward green products are strongly associated with increased desire. In the context of drone food delivery services, MGB-based research [44] evidences that attitude positively affects both desire and intention, while desire positively affects intention. Research on facial-recognition payments in the restaurant industry [45] demonstrates that among South Korean and American consumers, attitude positively influences both desire and intention, while desire has a significant positive effect on behavioral intention. Based on the aforementioned findings, the following hypotheses are proposed:

**H4.**  *Attitude positively influences desire toward HWF.*

**H5.**  *Attitude positively influences purchase intention toward HWF.*

**H6.**  *Desire positively influences purchase intention toward HWF.*

### 2.4. Measurement

The construct items derive from measurement scales used and prevalidated in existing literature and are evaluated using a five-point Likert scale ranging from “strongly disagree” (1) to “strongly agree” (5). The items were adapted from validated scales in previous research and were slightly modified to align with the context of this study. The research questionnaire was structured into five sections. Part One measured health-consciousness using a three-item scale adapted from Yadav and Pathak [46]. Part Two assessed attitude with four items based on Pang et al. [47]. Part Three evaluated desire, comprising four items derived from Perugini and Bagozzi [20] and Zhang et al. [48]. Part Four focused on purchase intention, employing a three-item scale adapted from Nystrand and Olsen [49]. Part Five collected respondents’ basic information, including gender, age, and education level. To ensure clarity and comprehension, the questionnaire was translated from English to Chinese using the back-translation method. Detailed information about the research constructs and questionnaire items is provided in Table A1.

### 2.5. Sample and Data Collection

For most individuals in China, the retirement age is 50 [50]. While individuals aged 50 or over may appear youthful, their physical abilities begin to decline [51]. Consistent with previous research, this study adopts 50 as the benchmark for defining older adults [52,53].

To address the issue of obtaining a complete sampling frame, this study employs a purposive sampling technique, a non-random sampling method that does not necessitate predefined hypotheses or a fixed sample size [54]. Purposive sampling enables researchers to deliberately select individuals who possess relevant knowledge or experiences and are willing to provide insightful responses [55]. To ensure adequate representation of the target population, this study focuses on individuals aged 50 to 65 with prior online shopping experience, residing in Zhejiang—a key region within the Yangtze River Delta. The Yangtze River Delta plays a crucial role in driving China’s economic growth and is among the earliest and most-developed hubs for e-commerce logistics in the country [56]. Additionally, the sampled individuals have a basic knowledge of HWF.

**Figure 2:**
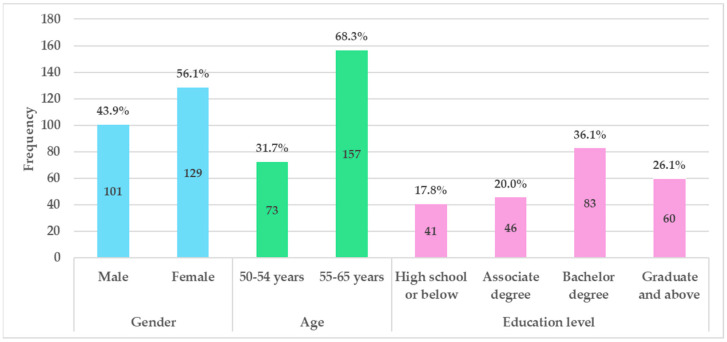
Demographic data of the respondents (*n* = 230).

Data were collected between November 2023 and March 2024 using online surveys via a Questionnaire Star platform on WeChat. This study was conducted in accordance with ethical standards, ensuring the confidentiality and anonymity of all respondents throughout the survey process. Informed consent was obtained from all participants, who were explicitly informed about the study’s purpose and their right to withdraw at any time. A total of 270 questionnaires were distributed, and 250 responses were collected. After excluding invalid samples due to response bias, such as those with no variance across questionnaire items or containing repeated answers, 230 valid samples were retained, resulting in a valid response rate of 85.2%. This response rate is considered acceptable, as supported by previous studies [57,58]. As outlined in [59], the ideal sample size is recommended to be a minimum of 10 times the number of items. With 14 items in the proposed model, the final sample size of this study substantially exceeds this threshold. Figure 2 provides the demographic information of the participants. Of the total respondents, 43.9% are male and 56.1% are female. Participants aged 50–54 account for 31.7%, while those aged 55–65 represent 68.3%. Additionally, the majority of participants hold a bachelor’s degree or higher (62.2%).

### 2.6. Data Analysis

The data analysis follows the guidelines for the integrated application of Partial Least Squares–Structural Equation Modelling (PLS–SEM) and Necessary Condition Analysis (NCA) [60,61]. PLS–SEM was employed for its robust predictive validity and explanatory power in the research model [62]. This method was used to estimate mediating and moderating effects, adhering to the recommendations of contemporary research [63,64]. The PLS–SEM analysis was conducted using a maximum iteration count of 3000, a tolerance level of 10^−7^, and a path weighting scheme. Additionally, percentile bootstrapping with 10,000 subsamples was applied to assess the relevance of structural model linkages, as suggested by Sarstedt et al. [62]. To evaluate the model’s predictive efficacy, cross-validated predictive ability test (CVPAT) was applied with 10 folds and 10 repetitions, in line with Sharma et al. [65]. Unstandardized latent variable scores derived from the PLS–SEM results were utilized for the NCA. Consistent with the recommendations of Richter et al. [27] and Hauff et al. [66], the ceiling envelopment–free disposal hull (CE-FDH) line was chosen for NCA due to the irregularity of data patterns near the ceiling lines, which achieve 100% accuracy. Furthermore, following Dul’s [67] method, 10,000 permutations were conducted to determine the significant levels of the effects. Data analysis was performed using SmartPLS 4 software.

## 3. Analysis and Results

### 3.1. Common Method Bias (CMB)

A solitary data source was used at one time point which necessitates consideration of the issue of common method bias (CMB). This study employs the unmeasured latent method construct test [68]. All substantive factor loadings in Table 1 are significant; however, the method loadings are not. The high 180:1 ratio of substantive to method variance suggests a minor impact from CMB. Furthermore, an analysis of the correlation coefficient matrix reveals the potential issue of CMB in the current data set. The correlation matrix indicates a maximum correlation of 0.676 between health-consciousness and desire, which is lower than the collinearity risk of 0.90 [69,70]. Therefore, CMB should most probably not be of concern in this research.

### 3.2. Measurement Results

The assessment model’s reliability and validity are established with factor loadings exceeding 0.708 [71], Cronbach’s alpha and composite reliability (CR) values exceeding 0.7, and all constructs’ average variance extracted (AVE) value exceeding 0.5 [72] (Table 2). All the heterotrait-monotrait (HTMT) ratio of correlations are below 0.90 (Table 3) [73]. The outcomes illustrate the measurement model demonstrates good reliability, and convergent and discriminant validity [65].

### 3.3. Path Analysis

**Figure 3:**
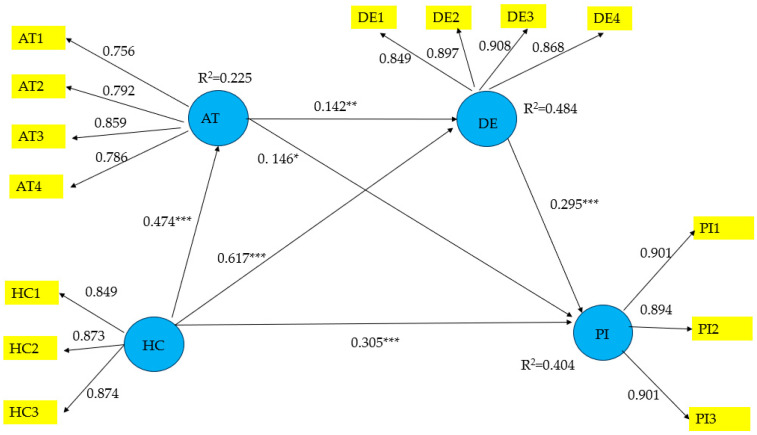
Structural equation modeling diagram. Source: Author’ own calculations using SmartPLS 4 software. Note: AT = attitude; DE = desire; HC = health-consciousness, PI = purchase intention. * *p* < 0.05; ** *p* < 0.01; *** *p* < 0.001.

According to the recommendations set forth by Hair et al. [74], the structural model hypotheses are subjected to evaluation through the implementation of a bootstrapping procedure with 5000 bootstrap resamples and a 95% bias-corrected and accelerated confidence interval. The results show all hypotheses are supported (Figure 3 and Table 4). Health-consciousness significantly affects attitude (β = 0.474, *p* < 0.001), desire (β = 0.617, *p* < 0.001), and purchase intention (β = 0.305, *p* < 0.001), verifying H1, H2, and H3. Attitude positively affects desire (β = 0.142, *p* < 0.01) and purchase intention (β = 0.146, *p* < 0.05), validating H4 and H5. Desire has a positive influence on purchase intention (β = 0.295, *p* < 0.001), confirming H6. The model explains 40.4% of the variance in purchase intention, 22.5% in attitude, and 48.4% in desire.

To enhance the internal validity of the study and mitigate the influence of confounding and extraneous factors, gender, age, and education were included as control variables in the analysis. The results indicate no significant relationship between these variables and purchase intention toward health and wellness food (HWF), suggesting that these demographic factors do not substantially impact consumers’ purchase intention toward HWF.

Further analysis was conducted with *f^2^* effect sizes to determine the explanatory power and the strength of the associations. Collectively, health-consciousness, attitude, and desire account for over 40% of the variance in purchase intention (*R^2^* = 0.404). Table 4 shows health-consciousness has the greatest effect on desire, exceeding the 0.35 threshold for a large effect size. Health-consciousness has a moderate impact on desire, surpassing the 0.15 threshold. Other paths, however, exhibit only minor effects.

Lastly, the fitness and predictive power of the model were assessed. The results demonstrate the standardized root mean square residuals (SRMR) are 0.059, which is below the threshold of 0.080 [75]. Furthermore, the PLSpredict procedure [76] reveals all three-purchase intention indicators have *Q*^2^ predict values above 0, and the root mean squared error (RMSE) of PLS–SEM is below the naïve linear model benchmark. These outcomes collectively indicate the model exhibits strong predictive capacity. In order to verify the model’s predictive performance, an analysis of the outcomes from the cross-validated predictive ability test (CVPAT) was conducted. The CVPAT method, as introduced by Sharma et al. [65] and Liengaard et al. [77], was utilized to evaluate the model’s construct and overall predictive capabilities. The findings show the model significantly lowers the naïve indicator averages and conservative linear model benchmarks, confirming the model’s predictive strength. The results from the PLSpredict and CVPAT, as shown in Table 5, collectively reveal the model’s predictive capability.

### 3.4. Analyzing Mediating Effects

The sequential mediation models were examined using SmartPLS 4. The calculation of indirect effects was undertaken utilizing a bias-corrected bootstrapping procedure with a sample size of 5000, employing random sampling. The presence of a 95% bias-corrected confidence interval (BCCI) that does not include zero indicates a significant mediation effect. As shown in Table 4, the indirect effect path (HC → AT → PI) is statistically significant, suggesting that attitude partially mediates the relationship between health-consciousness and purchase intention. Similarly, the indirect effect path (HC → DE → PI) is significant, indicating that desire also partially mediates the relationship between health-consciousness (HC) and purchase intention (PI). Furthermore, the sequential mediation analysis reveals that the indirect effect path (HC → AT → DE → PI) in the sequential mediation model is significant. These results indicate attitude and desire act as sequential co-mediators in the HC-PI relationship. When health-consciousness increases, attitude also increases, which in turn increases desire, resulting in an increase in purchase intention.

### 3.5. Necessary Condition Analysis (NCA)

**Figure 4:**
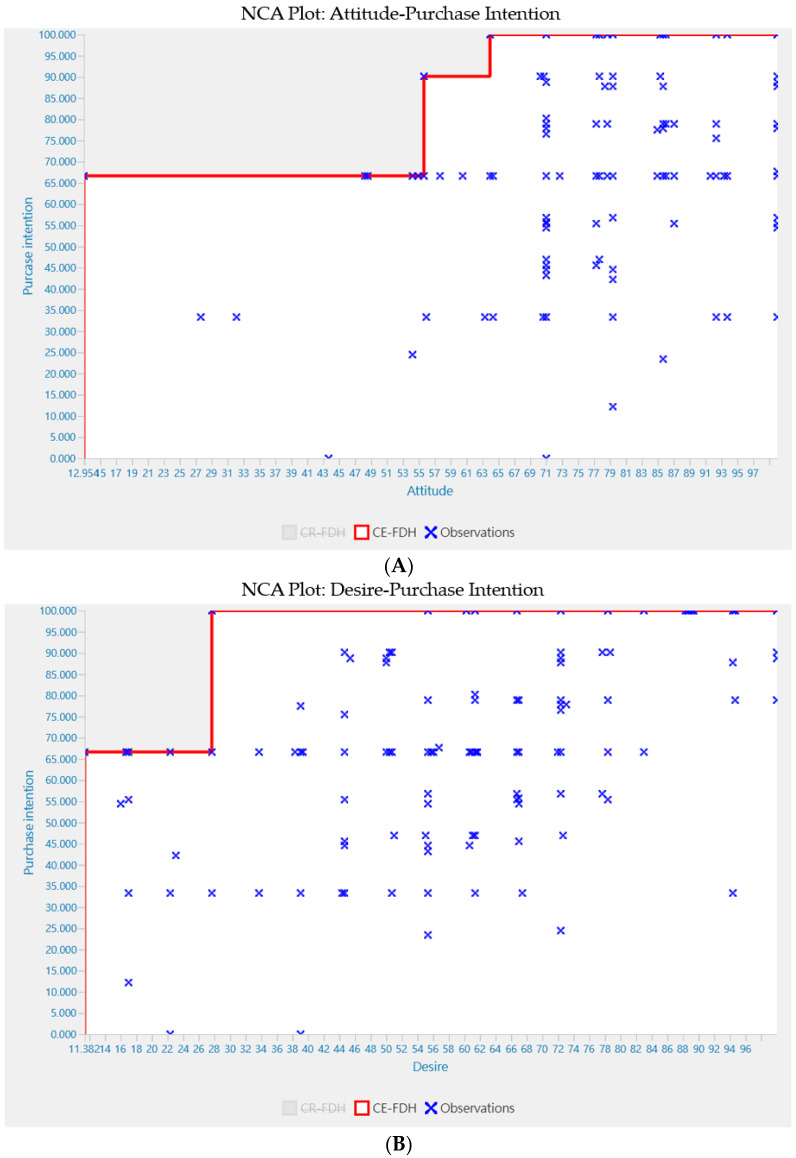

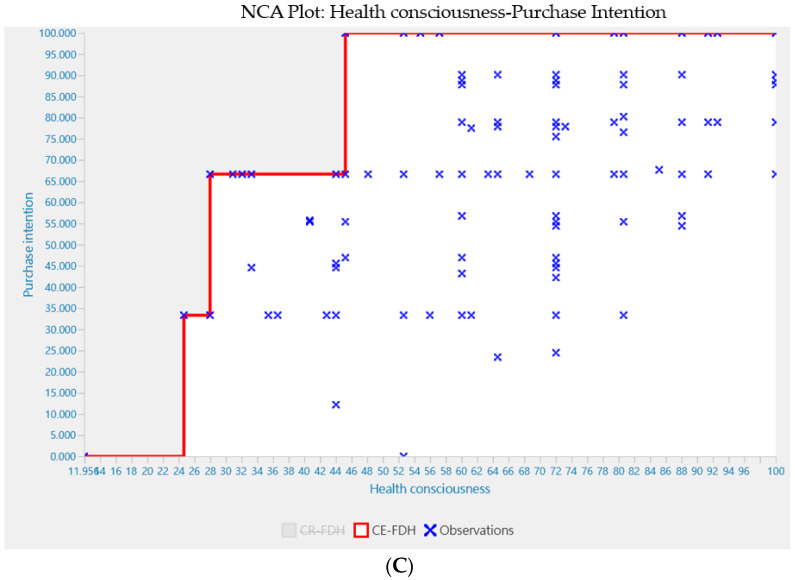
(**A**). NCA scatter plots with CE-FDH ceiling (attitude vs. purchase intention). (**B**). NCA scatter plots with CE-FDH ceiling (desire vs. purchase intention). (**C**). NCA scatter plots with CE-FDH ceiling (health-consciousness vs. purchase intention). Source: Author’ own calculations using SmartPLS 4 software.

After applying PLS–SEM, this study employed NCA [27] to ascertain the extent to which predictor variables are necessary for purchase intention. NCA is regarded as a pioneering approach for gauging the magnitude of “necessary, but not sufficient” effects exerted by predictor variables on outcome variables [78]. Proceeding to the NCA outcomes, visual scrutiny of the scatter plots is the initial step. Each scatter plot’s upper left area remains blank, indicating the likelihood of necessary conditions (Figure 4). Furthermore, there are no scatter plots showing significant ceiling (ceiling outliers) or scope (scope outliers).

The necessity effect sizes (*d*) were examined in terms of their significance and size in relation to the target construct of purchase intention. In line with the approach set out by Richter et al. [27] and Hauff et al. [66], this research refers to the ceiling-envelope free disposal hull (CE-FDH) ceiling line. The examination reveals health-consciousness and desire are necessary conditions for purchase intention, whereas attitude is not identified (Table 6).

To gain a deeper insight into the NCA findings, a bottleneck table was created. The critical value levels of the antecedent variable, which ensures the desired purchase intention level is achieved, is determined and outlined in Table 7. Based on the findings, an ambitious yet feasible target of 85 was established for purchase intention, with associated levels set at 56 for attitude, 28 for desire, and 46 for health-consciousness. Scoring 85 on the purchase intention scale (out of a possible 100) necessitates achieving 56 for attitude, 28 for desire, and 46 for health-consciousness. Table 8 illustrates the proportion of cases that are below the essential levels for the antecedent construct. For instance, 3.913% of cases do not reach the necessary attitude level, preventing them from reaching a purchase intention score of 85 (scale 0–100). Similar percentages for other antecedents are 4.783% for desire and 12.174% for health-consciousness. The following section will utilize this information to further analyze the initial Combined Importance-Performance Map Analysis (cIPMA) outcomes.

### 3.6. Combined Importance-Performance Map Analysis (cIPMA)

In an effort to gain deeper insights, the cIPMA framework was merged with NCA, a method proposed by Hauff et al. [66], to elucidate the critical factors for attaining purchase intention. The results illustrate the significance of constructs for purchase intention, as indicated by PLS–SEM total effects, and additionally demonstrate the mean rescaled latent variable scores of health-consciousness, attitude, and desire from SmartPLS 4 PLS–SEM analyses (Table 9). The percentage of cases that do not satisfy the essential threshold (specifically, cases with purchase intention scores below the necessary 85) are also revealed.

**Figure 5:**
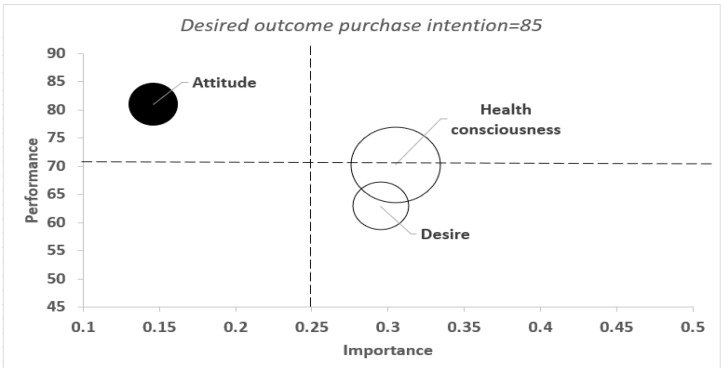
Combined importance–performance map. Source: Author’ own calculations using SmartPLS 4 software. Note: ○ = necessary condition construct; ● = not necessary condition construct; bubble size represents the proportion of cases that do not achieve the required level for the desired outcome.

The cIPMA analysis reveals 12.174% of cases are unable to reach the critical health-consciousness standard for purchase intention to achieve the target of 85, indicating a gap despite health-consciousness’s noted high relative importance and performance (Table 9). As indicated by the large bubble in Figure 5, health-consciousness holds a relatively higher importance and performance status. When contrasted with desire, a considerably smaller 4.783% of cases are unable to reach the critical desire level for purchase intention, reflected in the bubble’s smaller size.

## 4. Discussion

In comparison to previous research, the novelty of this study lies in its adoption of a multi-analytic approach, integrating Partial Least Squares–Structural Equation Modeling (PLS–SEM), Necessary Condition Analysis (NCA), and Combined Importance–Performance Map Analysis (cIPMA) to examine the impact of health-consciousness on purchase intention toward health and wellness food in China, with a particular focus on the mediating roles of attitude and desire. This study aims to distinguish between factors that serve as probabilistic causes of purchase intention and those that function as necessary conditions—critical factors or bottlenecks that must be present to achieve a certain level of purchase intention. Furthermore, the application of the novel cIPMA approach, which synthesizes probabilistic sufficiency and deterministic necessity logics, enhances the practical prioritization of managerial efforts and facilitates the targeted improvement of key constructs influencing purchase intention toward health and wellness food. This integrated methodological approach has rarely been used in existing research on health and wellness food consumption. The key findings are summarized as followed.

### 4.1. The Direct and Indirect Influences of Health-Consciousness, Attitude, and Desire

The findings of this study reveals consumers’ health-consciousness significantly influences their purchase intention toward health and wellness foods (HWF), both directly and indirectly. These findings align with prior research by Chen et al. [5] in China and Quededo-Silva et al. [8] in Brazil. The results also resonate with existing studies on organic food, which emphasize the critical role of health-consciousness in shaping consumers’ purchase intention toward organic food [37,39,79]. Furthermore, older adults (aged 50–65) in China are particularly inclined to purchase HWF due to their heightened health-consciousness. This trend is consistent with recent shifts in food-consumption patterns, as an increasing number of Chinese consumers opt for healthier meals [80]. In addition, health-consciousness directly influences attitude, corroborating findings from previous studies that health-consciousness significantly impacts attitude [36,38,39,81]. To cultivate a positive perception and attitude toward HWF, producers should emphazise the health benefits of HWF in promotional and advertising strategies.

Furthermore, the results highlight attitude as an importgant determinant of older adults’ purchaainf intention toward HWF, aligning with prior studies [4,5,30,82]. Additionally, attitude directly influences desire, supporting earlier research demonstrating the significant impact of attitude on desire [6,23,44]. Desire exerts both direct and indirect effects on purchase intention, specifically mediating the influence of health-consciousness on purchase intention, in conjunction with attitude. In other words, as health-consciousness and attitude increase, desire for HWF and purchase intention toward HWF rise correspondingly, indicating these motivational factors effectively predict desire and purchase intention. Consequently, marketing and promotional strategies should leverage the motivational factor, health-consciousness, to persuade non-HWF consumers and reinforce purchase behavior among existing HWF consumers. Furthermore, addressing concerns consumers may have regarding the perceived health benefits of HWF is essential for enhancing positive perceptions and motivating purchase intention.

### 4.2. The Serial Mediating Role of Attitude and Desire

The most significant finding of this study is the serial mediating role of attitude and desire in the indirect impact of health-consciousness on consumers’ purchase intention. This finding supports the theory that consumers with high health-consciousness are influenced by attitude and desire, which in turn motivates them to purchase HWF. Enhancing health-consciousness can encourage consumers to develop favorable attitudes, heighten desire, and ultimately a stronger willingness to purchase HWF. This study contributes to the expanding body of research on the influence of consumer awareness on behavior, as proposed by Williams and Poehlman [83]. Both attitude and desire have significant individual mediating effects and play a critical role as sequential mediators, amplifying the impact of health-consciousness on purchase intention. The sequential mediation analysis also reveals the significant mediating effect of attitude between health-consciousness and desire, as well as desire between attitude and purchase intention.

### 4.3. Multi-Analytic Approach (PLS–SEM, NCA, cIPMA) Enhance the Robustness of the Results

Overall, the PLS–SEM findings validate health-consciousness, attitude, and desire are substantial predictors of purchase intention, with attitude and desire serving as mediators. Consequently, growth in each construct contributes to Chinese older adults’ purchase intention toward HWF. The Necessary Condition Analysis results show the two constructs (health-consciousness and desire) play a crucial role in purchase intention. Yet, the bottleneck tables’ analysis reveals high levels of purchase intention can be achieved even with relatively low health-consciousness and desire levels. The Combined Importance–Performance Map Analysis results indicate facilitating purchase intention necessitates a focus on multiple constructs (health-consciousness and desire), particularly those deemed crucial for purchase intention but are currently linked to a high percentage of cases that do not meet the required standards.

## 5. Implications and Recommendations

### 5.1. Theoretical Implications

The results of this study have significant theoretical implications. First, the research model is grounded in a simplified perspective of the Model of Goal-Directed Behavior, investigating the direct impact of health-consciousness on purchase intention toward health and wellness foods (HWF) and its indirect effect through the sequential mediation of attitude and desire. Second, the methodology is strengthened by the integration of PLS–SEM, NCA, and cIPMA. This comprehensive approach is employed to explore the influence of health-consciousness, attitude, and desire on consumers’ purchase intention toward HWF. The integration of these methodologies enhances the robustness of the findings. Consequently, this comprehensive analytical framework is expected to provide a holistic understanding of consumers’ purchase intention toward HWF, thereby contributing to the growing body of consumer behavior literature in a HWF context.

### 5.2. Managerial Implications

This study offers several managerial implications. First, consumers with higher health-consciousness and a positive attitude toward HWF represent a loyal market segment. Effective segmentation strategies should be designed to target this group, enabling the development of tailored promotional strategies that resonate with their values and preferences. Second, although demand for HWF is increasing, many consumers remain unaware of the actual benefits and the impact of sustainable farming practices. To address this issue, comprehensive educational and promotional programs should be implemented to enhance consumer recognition of the health benefits associated with HWF. These campaigns should emphasize the different production methods used for both HWF and conventional foods, while highlighting the positive health impacts HWF provide. Moreover, advertisements can educate consumers on the reliability of HWF certifications, helping to build trust in the authenticity and quality of these products over time.

In conclusion, the utility of the study is to provide valuable insights into the factors influencing consumers’ purchase intention toward health and wellness food. The findings offer practical implications for organizations and managers seeking to optimize their marketing strategies and enhance sales performance. By understanding the roles of health-consciousness, attitude, and desire in shaping purchase intentions, companies can design targeted marketing campaigns that effectively resonate with their target audience, ultimately driving consumer engagement and fostering brand loyalty.

### 5.3. Limitations and Future Works

Alhough this study makes a substantial contribution to existing theories and practices on this topic, the results and findings must be interpreted while considering the following limitations.

First, the findings are elicited using a sample of adults aged 50–65 in Zhejiang, China, which may limit generalizability. Future research should consider broader demographics and geographical regions to enhance external validity. Second, a non-probability sample was used in the study, which may limit the representativeness of the results. To improve generalizability, future studies should use random sampling.

Third, this study employed a cross-sectional approach, meaning that data was collected at a single point in time. While significant relationships among health-consciousness, attitude, desire, and purchase intention were identified, caution is advised when making causal inferences. Future research could benefit from employing a longitudinal approach to better establish causal relationships.

Fourth, this study does not extensively discuss the role of social norms and cultural values, which may significantly influence consumer behavior toward health and wellness food. Future research could incorporate cultural and social dimensions to provide a more holistic understanding of consumer decisions.

Finally, this study adopts a dual methodological approach, integrating a symmetric perspective (PLS–SEM) with an asymmetric perspective (Necessary Condition Analysis), to comprehensively investigate the phenomenon. Future research is encouraged to explore the purchase intention of HWF using alternative methodologies, such as Artificial Neural Networks (ANN) and Fuzzy Set Qualitative Comparative Analysis (fsQCA).

## 6. Conclusions

This study aims to investigate the impact of health consciousness on purchase intention toward health and wellness food in China, with a particular emphasis on the serial mediating roles of attitude and desire. A multi-analytic approach is adopted, integrating Partial Least Squares-Structural Equation Modeling (PLS-SEM), Necessary Condition Analysis (NCA), and Combined Importance-Performance Map Analysis (cIPMA). The key findings indicate that health consciousness significantly influences purchase intention toward health and wellness food both directly and indirectly through attitude and desire. NCA results further demonstrate that health consciousness and desire serve as necessary conditions for purchasing health and wellness food, whereas attitude does not. This research contributes to the existing literature and theoretical understanding of consumer behavior while offering several important managerial implications for enterprise decision-makers.

## Figures and Tables

**Table 1:** Common method factor analysis.

Indicators	Substantive Factor Loading (*R_a_*)	Substantive Variance ($R_{a}^{2}$)	Method Factor Loading (*R_b_*)	Method Variance ($R_{b}^{2}$)
HC1	0.848 ***	0.719	0.126 *	0.016
HC2	0.874 ***	0.764	0.084 ^ns^	0.007
HC3	0.874 ***	0.764	0.075 ^ns^	0.006
AT1	0.754 ***	0.569	0.041 ^ns^	0.002
AT2	0.798 ***	0.637	0.001 ^ns^	0.000
AT3	0.855 ***	0.731	−0.007 ^ns^	0.000
AT4	0.788 ***	0.621	−0.129 ^ns^	0.017
DE1	0.853 ***	0.728	0.040 ^ns^	0.002
DE2	0.897 ***	0.805	−0.026 ^ns^	0.001
DE3	0.910 ***	0.828	−0.062 ^ns^	0.004
DE4	0.862 ***	0.743	−0.013 ^ns^	0.000
PI1	0.848 ***	0.719	0.002 ^ns^	0.000
PI2	0.874 ***	0.764	0.021 ^ns^	0.000
PI3	0.874 ***	0.764	0.012 ^ns^	0.000
Average	0.851	0.725	0.012	0.004

Source: Author’ own calculations using SmartPLS 4 software. Note: AT = attitude; DE = desire; HC = health-consciousness, PI = purchase intention; * *p* < 0.05; *** *p* < 0.001; ns denotes insignificant.

**Table 2:** Reliability and validity.

	Loading	Cronbach’s Alpha	Composite Reliability (rho_c)	Consistent Reliability Coefficient (rho_a)	Average Variance Extracted (AVE)
Suggested Cutt-Off Level	>0.708	>0.7	>0.7	>0.7	>0.5
Health-consciousness		0.832	0.899	0.833	0.749
HC1	0.849				
HC2	0.873				
HC3	0.874				
Attitude		0.811	0.876	0.824	0.639
AT1	0.756				
AT2	0.792				
AT3	0.859				
AT4	0.786				
Desire		0.903	0.932	0.907	0.775
DE1	0.849				
DE2	0.897				
DE3	0.908				
DE4	0.868				
Purchase intention		0.881	0.926	0.885	0.807
PI1	0.901				
PI2	0.894				
PI3	0.901				

Source: Author’ own calculations using SmartPLS 4 software. Note: AT = attitude; DE = desire; HC = health-consciousness, PI = purchase intention.

**Table 3:** Discriminant validity-TMT point estimates and confidence intervals.

	HC	AT	DE	PI
HC	-			
AT	0.573 [0.464, 0.666]	-		
DE	0.786 [0.707, 0.856]	0.502 [0.396, 0.596]	-	
PI	0.670 [0.572, 0.747]	0.490 [0.377, 0.593]	0.629 [0.536, 0.708]	-

Source: Author’ own calculations using SmartPLS 4 software. Note: AT = attitude; DE = desire; HC = health-consciousness, PI = purchase intention.

**Table 4:** Results of the overall path analysis of the model.

				Bias-Corrected and Accelerated Confidence Interval			
		β (Path Coefficient)	t-Value	5%	95%	Hypothesis Test	Effect *f*^2^
H1	HC → AT	0.474 ***	9.286	0.378	0.548	Supported	0.290
H2	HC → DE	0.617 ***	13.131	0.539	0.693	Supported	0.573
H3	HC → PI	0.305 ***	4.052	0.173	0.421	Supported	0.077
H4	AT → DE	0.142 **	2.683	0.049	0.225	Supported	0.030
H5	AT → PI	0.146 *	2.266	0.039	0.251	Supported	0.027
H6	DE → PI	0.295 ***	4.112	0.174	0.412	Supported	0.075
Indirect effect							
HC → AT → PI		0.069 *	2.195	0.019	0.122		
HC → DE → PI		0.182 ***	3.915	0.108	0.262		
HC → AT → DE → PI		0.020 *	2.116	0.008	0.039		
AT → DE → PI		0.042 *	2.173	0.016	0.080		
HC → AT → DE		0.067 **	2.627	0.025	0.110		

Source: Author’ own calculations using SmartPLS 4 software. Note: AT = attitude; DE = desire; HC = health-consciousness, PI = purchase intention. * *p* < 0.05; ** *p* < 0.01; *** *p* < 0.001.

**Table 5:** Explanatory power, PLSpredict, and CVPAT.

	Explanatory Power	Predictive Assessment: PLSpredict				Predictive Assessment: CVPAT	
	R^2^		*Q*^2^ Prediction	PLS–SEM RMSE	LM RMSE	Average PLS Loss-IA Loss (*p* Value)	Average PLS Loss- LM Loss (*p* Value)
AT	0.225	PI1	0.301	0.580	0.584	−0.060 (0.000)	−0.002 (0.633)
DE	0.484	PI2	0.236	0.654	0.661	−0.237 (0.000)	0.002 (0.812)
PI	0.404	PI3	0.236	0.599	0.605	−0.129 (0.000)	−0.007 (0.043)

Source: Author’ own calculations using SmartPLS 4 software. Note. IA = indicator-averages prediction benchmark; LM = linear model prediction benchmark; AT = attitude; DE = desire; PI = purchase intention.

**Table 6:** Necessity effect sizes (CE-FDH ceiling line).

	Sustainable Performance	
Construct	Effect Size *d*	*p*-Value
Attitude	0.173	0.107
Desire	0.061	0.038
Health-consciousness	0.235	0.000

Source: Author’ own calculations using SmartPLS 4 software. Note: 0 *< d <* 0.1 small effect, 0.1 < *d <* 0.3 medium effect, 0.3 *< d <* 0.5 large effect, and *d >* 0.5 very large effect [26].

**Table 7:** Bottleneck table of purchase intention, actual values (recalculated from PLS–SEM latent variable on a 0–100 scale).

Purchase Intention	Attitude	Desire	Health-Consciousness
0	NN	NN	NN
5	NN	NN	24.649
10	NN	NN	24.649
15	NN	NN	24.649
20	NN	NN	24.649
25	NN	NN	24.649
30	NN	NN	24.649
35	NN	NN	27.989
40	NN	NN	27.989
45	NN	NN	27.989
50	NN	NN	27.989
55	NN	NN	27.989
60	NN	NN	27.989
65	NN	NN	27.989
70	55.637	27.663	45.22
75	55.637	27.663	45.22
80	55.637	27.663	45.22
85	55.637	27.663	45.22
90	55.637	27.663	45.22
95	63.935	27.663	45.22
100	63.935	27.663	45.22

Source: Author’ own calculations using SmartPLS 4 software. Note: NN = Not necessary.

**Table 8:** Bottleneck table of purchase intention: actual values for purchase intention (based on the rescaled PLS–SEM latent variable scores from 0–100) and the percentiles of antecedent constructs.

Purchase Intention	Attitude		Desire		Health-Consciousness	
Actual Values (Rescaled 1–100)	Percentage (Number) of Cases Below the Stipulated Benchmarks					
0	0	(0)	0	(0)	0	(0)
5	0	(0)	0	(0)	0.435	(1)
10	0	(0)	0	(0)	0.435	(1)
15	0	(0)	0	(0)	0.435	(1)
20	0	(0)	0	(0)	0.435	(1)
25	0	(0)	0	(0)	0.435	(1)
30	0	(0)	0	(0)	0.435	(1)
35	0	(0)	0	(0)	0.87	(2)
40	0	(0)	0	(0)	0.87	(2)
45	0	(0)	0	(0)	0.87	(2)
50	0	(0)	0	(0)	0.87	(2)
55	0	(0)	0	(0)	0.87	(2)
60	0	(0)	0	(0)	0.87	(2)
65	0	(0)	0	(0)	0.87	(2)
70	3.913	(9)	4.783	(11)	12.174	(28)
75	3.913	(9)	4.783	(11)	12.174	(28)
80	3.913	(9)	4.783	(11)	12.174	(28)
85	3.913	(9)	4.783	(11)	12.174	(28)
90	3.913	(9)	4.783	(11)	12.174	(28)
95	7.391	(17)	4.783	(11)	12.174	(28)
100	7.391	(17)	4.783	(11)	12.174	(28)

Source: Author’ own calculations using SmartPLS 4 software.

**Table 9:** cIPMA results.

Antecedent Construct	Importance	Performance	Percentage of Cases That Do Not Meet the Necessity Condition ^a^	Necessity Effect Size of *d* (*p* Value)
AT	0.146	81.016%	3.913%	0.173 (0.107)
DE	0.295	62.958%	4.783%	0.061 (0.038)
HC	0.305	70.256%	12.174%	0.235 (0.000)

Source: Author’ own calculations using SmartPLS 4 software. Note: AT = attitude; DE = desire; HC = health-consciousness; ^a^ based-on purchase intention performance outcome level of 85%.

## Data Availability

The data presented in this study are available on request from the corresponding author due to privacy.

## Appendix A

**Table A1:** Study variables and items with means, standard deviations, skew, and kurtosis.

Study Variables and Items	Mean	SD	Skew	Kurtosis
Health-consciousness (HC)				
HC1. I choose food carefully to ensure good health.	4.09	0.719	−1.056	2.433
HC2. As I go about my day, I am conscious of the state of my health.	3.92	0.822	−0.807	0.723
HC3. I often think about health-related issues.	3.84	0.715	−0.332	0.099
Attitude				
AT1. Buying a health and wellness food benefits my health.	4.42	0.627	−1.563	6.576
AT2. Buying a health and wellness food benefits my appearance.	4.27	0.630	−0.909	2.496
AT3. Purchasing health and wellness food is a good idea.	4.25	0.733	−1.231	2.686
AT4. I have a favorable attitude toward purchasing health and wellness food.	4.44	0.623	−0.989	1.508
Desire (DE)				
DE1. I want to purchase health and wellness food for better health.	3.43	0.857	−0.104	−0.478
DE2. I wish to purchase health and wellness food for health benefits.	3.72	0.836	−0.386	−0.098
DE3. I am eager to purchase health and wellness food to attain health goals.	3.46	0.894	−0.330	−0.304
DE4. My wish to purchase health and wellness food ensures my state of health.	3.85	0.690	−0.522	0.614
Purchase intention (PI)				
PI1. I intend to eat health and wellness food regularly.	4.10	0.691	−0.380	−0.045
PI2. I expect to eat health and wellness food regularly.	3.97	0.747	−0.591	0.436
PI3. I plan to eat health and wellness food regularly.	4.07	0.683	−0.420	0.280

Note: SD = standard deviation.
